# Case Report: Hereditary spastic paraplegia associated with monoallelic variant in the motor domain of KIF1A

**DOI:** 10.3389/fnhum.2025.1568511

**Published:** 2025-05-19

**Authors:** Kathryn Sine, David Brodie-Mends, Wafae Chouhani, Lauren Massingham, Saud Alhusaini

**Affiliations:** ^1^Department of Neurology, Alpert Medical School at Brown University, Providence, RI, United States; ^2^Division of Genetics, Department of Pediatrics, Hasbro Children’s Hospital, Providence, RI, United States; ^3^Division of Genetics, Department of Pediatrics, Alpert School of Medicine at Brown University, Providence, RI, United States; ^4^Movement Disorders Program, Rhode Island Hospital, Brown University Health, Providence, RI, United States

**Keywords:** familial case report, neurogenetics, hereditary spastic paraplegia, kinesin superfamily motor protein, KIF1A

## Abstract

**Objectives:**

To investigate the genetic etiology of a familial case with spastic paraplegia.

**Methods:**

Neurological examination, clinical and genetic work-up, including exome sequencing (ES), followed by targeted testing, were performed to determine the underlying etiology of the patients’ phenotype.

**Results:**

A 45-year-old man was initially diagnosed with spastic diplegic cerebral palsy in early childhood. He underwent multiple orthopedic interventions for lower extremities spasticity and progressive gait disturbance. His son developed similar neurological symptoms at 2-years of age. Despite unremarkable initial work-up, their relatively similar slowly progressive phenotype was suggestive of hereditary spastic paraplegia (HSP). ES was performed for the son at age 11 years, followed by cascade single testing for the father, which revealed a heterozygous (monoallelic) likely pathogenic variant [NM_001244008.2: c.947G > A (p.Arg316Gln); chr2-240775862] in exon 10 of the *KIF1A* gene.

**Discussion:**

*KIF1A* codes for a kinesin-3 motor protein involved in neuronal axon vesicular transport. *KIF1A* pathogenic variants are associated with several neurological phenotypes, most commonly HSP. The rare likely pathogenic variant (p.Arg316Gln) reported here was associated with an autosomal dominant HSP with few complications.

## Introduction

Hereditary spastic paraplegia (HSP) refers to a clinically heterogeneous group of inherited conditions characterized by degeneration of the corticospinal tracts and dorsal columns (Shribman et al., 2019; Meyyazhagan et al., 2022). The incidence of HSP is approximately 1–5 per 100,000 individuals (Pennings et al., 2020). HSP presents with progressive and disabling lower extremities spasticity and weakness, and based on the overall phenotype, it can be classified into “pure” or “complicated” forms (Shribman et al., 2019; Meyyazhagan et al., 2022). In contrast to the classic “pure” HSP, which is defined by a slowly progressive lower extremity spastic paraparesis, “complicated” HSP presents with additional neurological or systemic findings, such as seizures, cognitive and neurobehavioral symptoms, peripheral neuropathy, and optic atrophy (Montenegro-Garreaud et al., 2020). Several inheritance patterns have been observed, including autosomal dominant (AD), autosomal recessive (AR), X-linked, or mitochondrial (Shribman et al., 2019). Over 87 genetic loci have been associated with HSP, emphasizing its large genetic heterogeneity (Pennings et al., 2020). Using a targeted multigene panel, a study of 1,550 index cases with HSP identified a causative variant in only 30.7% of cases. Subsequent whole exome sequencing of 42 cases negative for the targeted panel increased the diagnosis rate to about 50% (Méreaux et al., 2022). This indicates that many genetic variants remain undiscovered.

In this report, we describe the clinical phenotype and work-up findings of a familial case of HSP with few complications related to a monoallelic likely pathogenic variant within the kinase motor domain of the kinesin family member 1A (*KIF1A*) gene (Lee et al., 2015; Ohba et al., 2015).

## Case description

Case 1 is a 45-year-old White male who was previously diagnosed with spastic diplegic cerebral palsy. Despite meeting all developmental milestones at an appropriate age, he developed progressive gait disturbance and stiffness of the lower extremities in early childhood. He was able to ambulate independently through late childhood. During adolescence, he underwent multiple orthopedic surgeries, including bilateral Achilles tendon lengthening, to improve lower extremities stiffness and ankle joints’ range of motion. His cognitive development was normal. He completed third-level education and is currently working as a special-needs teacher. More recently, he developed sphincter dysfunction, including urinary frequency and occasional stool incontinence.

**Figure 1:**
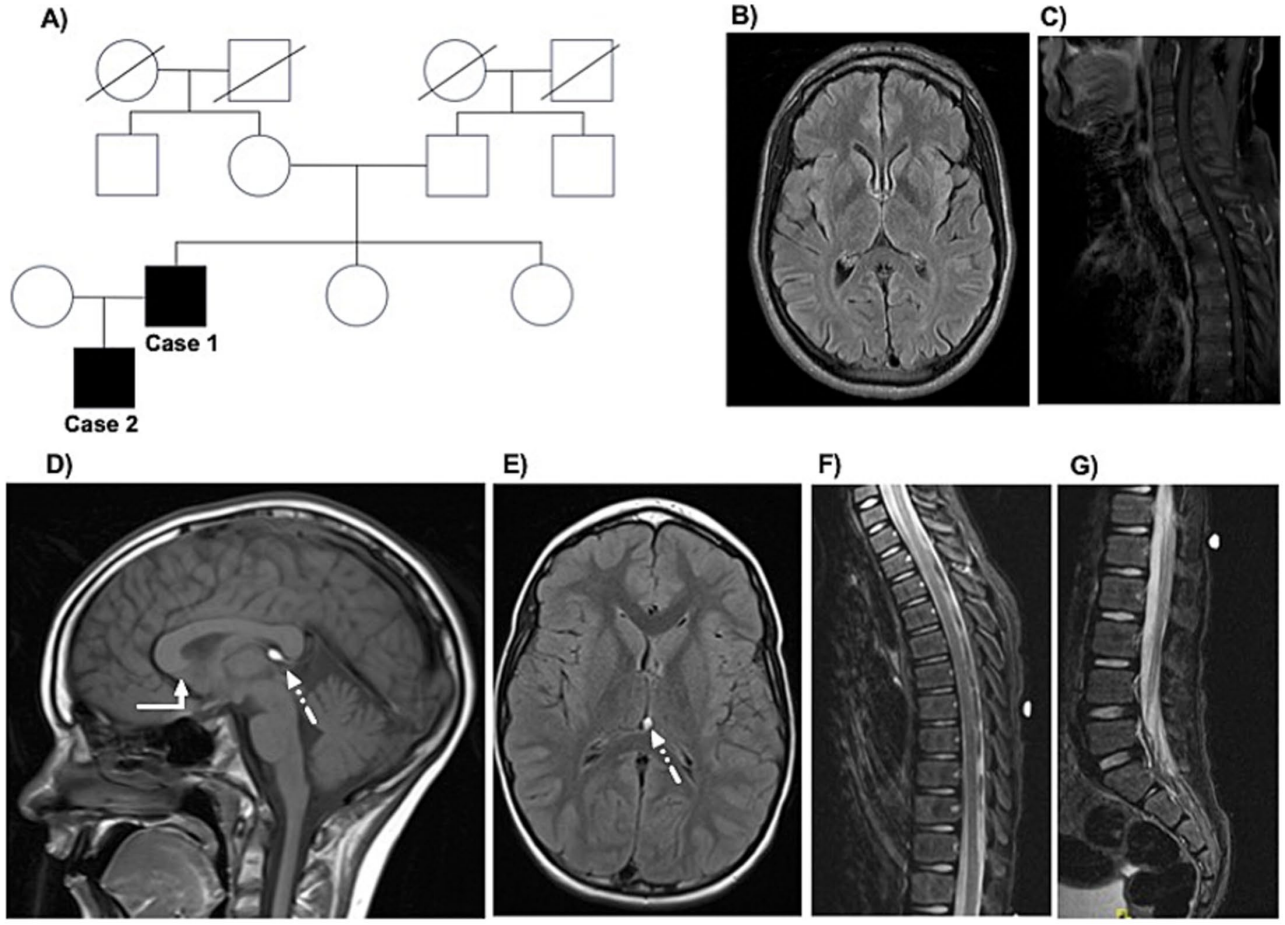
The family tree and MRI findings in the father-son cases. The family tree is displayed in panel **A**. Parts of the magnetic resonance imaging (MRI) of Case 1 obtained at age 44 at the initial movement disorders clinic appointment are displayed, including axial FLAIR sequences of the brain (panel **B**) and upper spine (panel **C**). Selected images of the MRI brain and spine of Case 2 obtained at age 8 at initial child neurology appointment are displayed, including sagittal T1-weighted (panel **D**; white arrow refers to the lack of visualization of the rostrum and mildly dysmorphic corpus callosum; dotted arrow refers to the 6-mm lipoma in the cavum vellum interpositum) and axial FLAIR sequences (panel **E**) of the brain and sagittal short tau inversion recovery (STIR) sequences of the upper and lumbar spine (panels **F,G**).

He reported no neurological symptoms or gait impairment in three generations of his family (see Figure 1A). He has two healthy younger sisters, aged 38 and 43. However, his son developed similar progressive neurological symptoms at age 2 (see Case 2).

His neurological examination at age 45 years was notable for spasticity, weakness (ranging from 3–4 out of 5 per the Medical Research Council manual muscle testing scale), and hyperreflexia in the bilateral lower extremities, with positive Babinski sign bilaterally. Impaired vibration and proprioception sensation was detected at the toes bilaterally. His gait was spastic. During work-up, he underwent an MRI of the brain and total spine, which were largely unremarkable (see Figures 1B,C). A formal neuropsychology evaluation revealed intact performance across all cognitive domains. For symptomatic management of spasticity, he is receiving Baclofen 20 mg twice daily. He also started chemo-denervation of the lower extremity muscles with botulinum toxin.

Given the development of similar neurological symptoms in his son, genetic testing was pursued (see detail below).

Case 2 is an 11-year-old White male who was born at full-term (38 weeks of gestation) by elective Cesarian section to a healthy 28-year-old primigravida mother. His birth weight was 6 lbs. 15 oz. The pregnancy was unremarkable, and the only documented perinatal complications were mild jaundice and a weak suck-and-swallow reflex, both of which were managed conservatively and resolved without further intervention. All of his early motor and speech-language developmental milestones were met at the expected age. However, at two years of age, he developed “toe walking.” This progressed to significant gait disturbance and recurrent falls. His initial neurological, metabolic, and genetic work-up was unrevealing. MRI of the brain revealed a mildly dysmorphic corpus callosum without visualization of the rostrum and 6-mm lipoma in the cavum vellum interpositum (see Figures 1D,E). Around age 5, he began to experience significant stiffness in the lower extremities. He later developed nocturnal enuresis, which raised concern for a possible learning disability. A neurodevelopment assessment led to a diagnosis of attention-deficit/hyperactivity disorder (ADHD), impulsivity, and mild executive dysfunction. At 8 years old, based on MRI spine findings (see Figures 1F,G), he was diagnosed with a possible tethered spinal cord syndrome and underwent a release procedure, with some reported gait improvement.

His neurological examination at 11 years of age was notable for spasticity and hyperreflexia in the lower extremities and bilateral weakness of the ankle dorsiflexion. His gait was spastic, with a notable toe walking. For symptomatic management, he received intensive physical therapy and required ankle-foot orthoses and later began chemo-denervation treatment with botulinum toxin for lower extremity spasticity management.

Exome sequencing (ES) was pursued at age 11. This revealed a likely pathogenic heterozygous (monoallelic) variant in exon 10 of *KIF1A* [NM_001244008.2: c.947G > A; (p.Arg316Gln); chr2-240775862]. His father (Case 1) underwent *KIF1A* gene sequencing at age 45, revealing the same likely pathogenic variant, confirming that this rare variant (which at the time of submission was not available on varsome.com) is linked to the hereditary spastic paraplegia phenotype in the father and son (Kopanos et al., 2019).

## Discussion

Given the genotype–phenotype heterogeneity in HSP, identifying the genetic etiology in many cases with suspect HSP phenotype is becoming increasingly reliant on advanced next-generation sequencing (NGS) technologies, such as ES (Shribman et al., 2019; Meyyazhagan et al., 2022; Méreaux et al., 2022). *KIF1A* encodes for a kinesin-3 motor protein that is highly expressed in the central nervous system (Gabrych et al., 2019). Kinesins are a diverse group of microtubule-based motor proteins that perform various functions, including intracellular transport of vesicles, organelles, chromosomes, and protein complexes (Lawrence et al., 2004). Originally, they were classified according to their specific functions and other criteria (e.g., the position of the motor core within the protein) (Lawrence et al., 2004). However, at the 2003 meeting of the American Society for Cell Biology, a dedicated subgroup of experts recommended a standardized nomenclature for all kinesins, grouping them into 14 superfamilies according to molecular phylogenetic analysis (Lawrence et al., 2004). Pathogenic variants in the *KIF1A* gene have been implicated in many neurodevelopmental and neurodegenerative disorders with diverse impacts on the central and peripheral nervous systems. *KIF1A* pathogenic variants have been associated with four disorders in Online Mendelian Inheritance in Man (OMIM) (Nicita et al., 2021). Two disorders are spastic paraplegia-30 (SPG30) due to pathogenic variants in *KIF1A* of either AR and AD inheritance patterns with overlapping phenotypes (Krenn et al., 2017; Lo Giudice et al., 2014). The third disorder, neurodegeneration and spasticity with or without cerebellar atrophy or cortical visual impairment (NESCAV syndrome), formerly named Mental Retardation Type 9 (MRD9), is a complex AD condition (Paprocka et al., 2023). NESCAV syndrome is characterized by childhood onset of moderate to severe intellectual disability with a broad range of other clinical features, which may represent an early onset of complicated HSP phenotype (Nicita et al., 2021). The fourth disorder is hereditary sensory and autonomic neuropathy type 2 (HSAN2), which has an AR inheritance pattern (Nicita et al., 2021). *KIF1A* variants have also been linked with progressive encephalopathy with brain atrophy, progressive neurodegeneration, PEHO (progressive encephalopathy with edema, hypsarrhythmia, optic atrophy)-like syndrome, and Rett-like syndrome (Paprocka et al., 2023).

**Figure 2:**
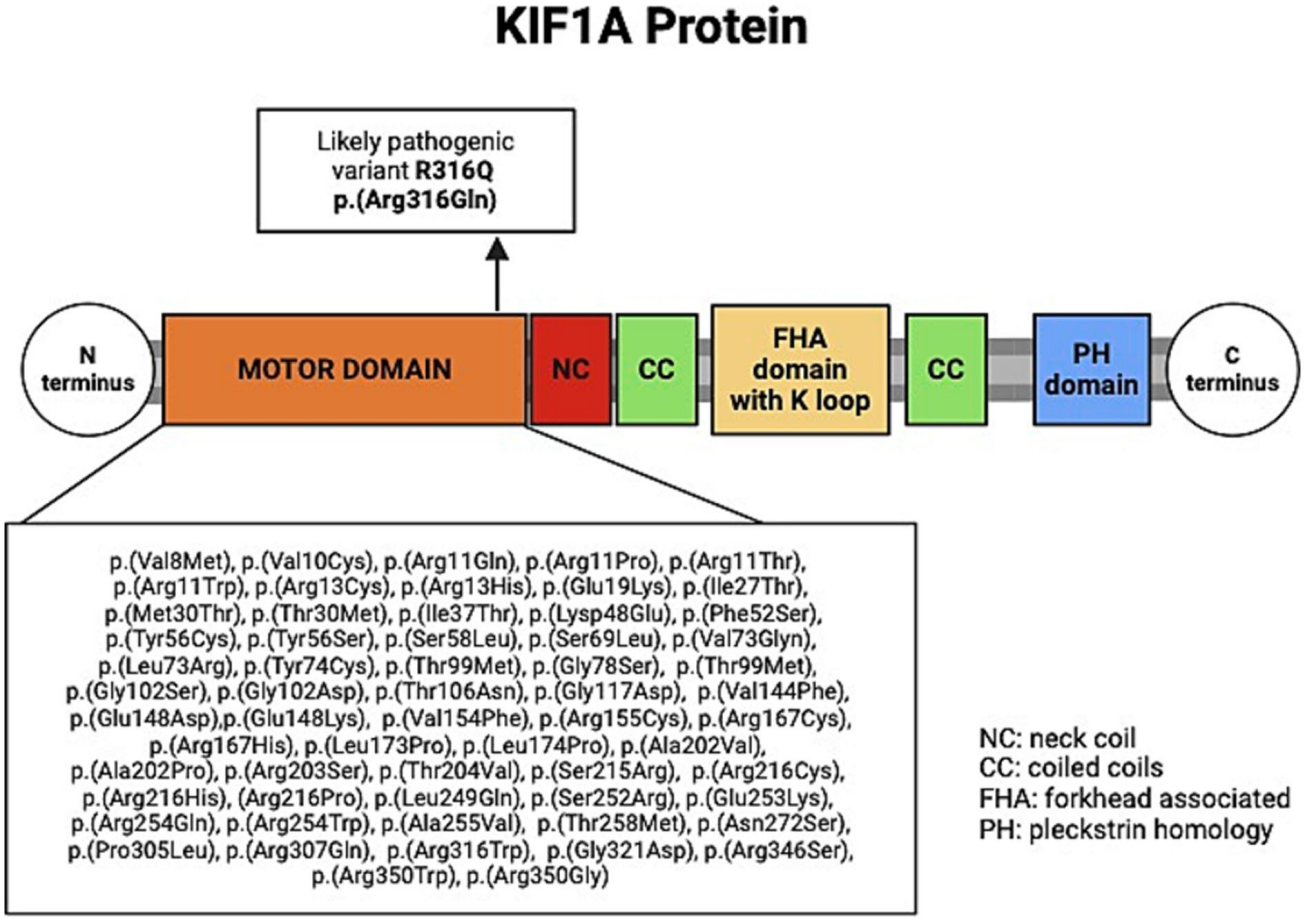
Schematic of the KIF1A protein, showing previously reported variants within the motor domain in *KIF1A*-related HSP. Created by BioRender.

Most HSP-related *KIF1A* variants are missense variants in the kinesin-3 motor domain of the KIF1A protein (residues 1 to 365, see Figure 2), and are often associated with “pure” or “complicated” HSP phenotype with *de novo* or AD inheritance (see Table 1) (Shribman et al., 2019; Montenegro-Garreaud et al., 2020; Lee et al., 2015). In this report, we describe the phenotype of a familial case with HSP due to a likely pathogenic monoallelic variant in exon 10 of the *KIF1A* gene. The same *KIF1A* variant, p.Arg316Gln, and another missense variant at the same residue, p.Arg316Trp, were previously reported in individuals with “complicated” HSP (Lee et al., 2015; Ohba et al., 2015). In the report by Hsu et al., carriers of the p.Arg316Gln variant exhibited axonal sensorimotor polyneuropathy and abnormal MRI findings, including thinning of the posterior corpus callosum and atrophy of the thoracic cord (Hsu et al., 2022). In contrast, the p.Arg316Gln variant identified in our cases appears to be associated with a slowly progressive “pure” HSP phenotype with few complications.

**Table 1:** Previously reported variants in the motor domain of KIF1A and their associated phenotypes and disorders.

KIF1A variant	Inheritance pattern	Associated phenotype(s)	Reference
p.Met30Thr, p.Tyr56Cys^+^, p.Ser69Leu^+^, p.Tyr74Cys, p.Gly78Ser, p.Thr106Asn, p.Arg167His^+^, p.Leu173Pro, p.Ser252Arg, p.Thr258Met, p.Arg350Trp^+^	AD (inherited)	Pure HSP phenotype Sensory disturbances in the lower or upper limbs Sphincter problems	Pennings et al. (2020)
p.Lys48Glu, p.Thr99Met^+^, p.Gly117Asp., p.Leu174Pro, p.Ala202Val, p.Arg216Cys^+^, p.Arg216His^+^, p.Asn272Ser^+^, p.Arg307Gln^+^, Arg316Trp^+^,	AD (*De novo*)	Complicated HSP phenotype Early onset cerebellar ataxia and cerebellar atrophy Variable degree of cognitive impairment Seizures Peripheral neuropathy	Nicita et al. (2021)
p.Arg216Cys^+^, p.Arg254Trp^+^, p.Pro305Leu^+^	AD (*De novo*)	Ataxic phenotype: Congenital ataxia Variable degree of intellectual disability Cerebellar atrophy Growth hormone deficiency Optic atrophy	Nicita et al. (2021)
p.Glu19Lys, p.Arg316Gln	AD (inherited)	Complicated HSP phenotype Axonal sensorimotor polyneuropathy Thoracic cord atrophy, thin corpus callosum and white matter hyperintensity.	Hsu et al. (2022)
p.Gly321Asp	AD (inherited)	Pure HSP phenotype	Hsu et al. (2022)
p.Arg13His, p.Val73Gly, p.Thr99Met^+^, p.Glu148Lys, p.Arg203Ser, p.Arg216Cys^+^, p.Asn272Ser^+^, p.Arg316Trp^+^	AD (*De novo*)	Complicated HSP phenotype Developmental delay Seizures Peripheral neuropathy Optic nerve atrophy Hip subluxation NESCAV	Paprocka et al. (2023)
p.Ser58Leu^+^, p.Thr99Met^+^, p.Gly102Asp., p.Val144Phe, p.Arg167Cys^+^, p.Ala202Pro, p.Ser215Arg, p.Arg216Pro, p.Leu249Gln, p.Glu253Lys^+^, p.Arg316Trp^+^	AD (*De novo*)	Complicated HSP phenotype Developmental delay Microcephaly Ataxia Limb spasticity Peripheral neuropathy Cerebellar atrophy Optic nerve atrophy	Lee et al. (2015)
p.Glu148Asp., p.Arg254Gln, p.Arg254Trp^+^, p.Arg307Gln^+^, p.Arg316Trp^+^	AD (*De novo*)	Complicated HSP phenotype Developmental delay Peripheral neuropathy Ataxia Cerebellar atrophy Optic nerve atrophy	Ohba et al. (2015)
p.Thr99Met^+^, p.Arg216Cys^+^, p.Arg216His^+^, p.Glu253Lys^+^, p.Arg316Trp^+^	AD (*De novo*)	Complicated HSP Global developmental delay Microcephaly Hypotonia Cerebellar atrophy	Esmaeeli Nieh et al. (2015)
p.Thr204_Val205delinsLys-Ala, p.Arg155Cys, p.Arg316Trp^+^	AD (*De novo*)	Ataxic phenotype: hypotonia and motor incoordination, progressive ataxia Seizures Developmental delay Language impairment Peripheral neuropathy Optic atrophy MRI abnormalities: cerebellar atrophy, corpus callosum involvement, white matter and dentate nuclei signal abnormality	Vecchia et al. (2022)
p.Ser69Leu^+^, p.Gly102Ser^+^, p.Arg167Cys^+^	AD (67% *De novo*)/ (33% inherited)	Pure and complicated HSP	Citterio et al. (2015)
p.Val8Met, p.Ile27Thr	AD (inherited)	Pure HSP	Iqbal et al. (2017)
p.Ile37Thr	AD (inherited)	Pure HSP	Dong et al. (2018)
p.Ala255Val	AR	Pure HSP	Erlich et al. (2011)
p.Arg11Thr	AD (*De novo*)	Complicated HSP Early Developmental delay Peripheral neuropathy	Morais et al. (2017)

^+^Variant is reported ≥ 1.

As evident in the reported cases of HSP-related *KIF1A* variants (see Table 1), the phenotypic heterogeneity associated with these variants likely represents a spectrum of HSP rather than many disorders converging at a single locus (Montenegro-Garreaud et al., 2020). Therefore, the presence of other neurological symptoms and findings in cases with classic HSP phenotype should warrant additional work-up and expert evaluation as clinically indicated. For example, the presence of visual symptoms or optic nerve atrophy should warrant an ophthalmologic evaluation. Similarly, neurocognitive testing, electroencephalography, and nerve conduction studies should be considered in patients with cognitive symptoms, concerns about a seizure disorder, or neuropathy symptoms. The AD inheritance is important for consideration of recurrence risk in preconception discussions.

Genetic identification of HSP variants is crucial and will guide the search for molecular targets for future disease-modifying therapies.

## Data Availability

The original contributions presented in the study are included in the article/supplementary material, further inquiries can be directed to the corresponding author/s.
